# Patients’ Perceptions of the Role of Nursing in Substance Use Disorder Treatment Programs: Qualitative Study

**DOI:** 10.2196/82401

**Published:** 2026-03-31

**Authors:** Clara Lucas Guerra, Marina Gómez de Quero Córdoba, Bruno García Tardón, Guadalupe González Naranjo, Noemí Mayoral Gonzalo

**Affiliations:** 1 Faculty of Nursing, Physiotherapy and Podiatry, Complutense University of Madrid, C/Emiliano Barral, 18A, 2º3, Madrid, 28043, Spain, 34 689593725; 2 Faculty of Health Sciences - HM Hospitals, Camilo Jose Cela University, Villanueva de la Cañada, Madrid, Spain; 3 HM Hospitals Health Research Institute, Madrid, Spain; 4 Research Group on Advanced Nursing (CARING)-161, Rovira i Virgili University, Tarragona, Spain; 5 Faculty of Education, Camilo Jose Cela University, Madrid, Spain

**Keywords:** nurse's role, nursing staff, qualitative research, substance-related disorder, psychiatric nursing

## Abstract

**Background:**

Substance use disorders are chronic conditions with significant personal and social consequences. Nursing care plays a key role in outpatient and community-based rehabilitation programs, yet patients’ perspectives on this role remain underexplored.

**Objective:**

This study aimed to explore how patients with substance use disorders perceive and interpret nursing care in community addiction treatment centers operated by the Spanish Red Cross in Madrid, Spain. Specifically, it sought to describe the organizational and care-related roles attributed to community addiction treatment centers and analyze patients’ perceptions of nurses’ technical and relational functions.

**Methods:**

A phenomenological qualitative design was used. Fourteen in-depth, semistructured interviews were conducted with patients undergoing treatment at Red Cross centers. Participants were selected through purposive sampling to ensure diversity in age, gender, substance use, and treatment experiences. The data were analyzed using systematic text condensation and supported by the ATLAS.ti software.

**Results:**

The center is perceived as a significant space not only for offering healthy leisure activities outside the context of substance use but also as a supportive environment that fosters a sense of belonging to a community. Patients valued the emotional support, empathy, and relational care provided by nurses, often highlighting their role in building trust and offering personalized attention. However, there was limited awareness of nurses’ technical competencies.

**Conclusions:**

These findings underscore the importance of holistic, patient-centered care and the need to enhance the visibility and recognition of nursing roles in addiction treatment settings.

## Introduction

### Background

Substance use disorders (SUDs) are defined by the *DSM-5* (*Diagnostic and Statistical Manual of Mental Disorders, Fifth Edition*) as the “association of cognitive, behavioral, and physiological symptoms that indicate that the person continues to use a substance despite significant problems related to that substance” [1]. Globally, substance use represents a major public health concern and contributes substantially to the burden of disease, being associated with increased mortality, disability, and chronic conditions, such as liver disease, cancer, and infectious diseases [2-4]. SUDs commonly coexist with psychiatric disorders, particularly mood and anxiety disorders, which complicate treatment and worsen prognosis [3,5,6]. Consequently, SUDs can have a profound impact on a person’s identity, causing individuals to lose their sense of self, which can manifest itself as a change in values and priorities, a deterioration of self-image, social disconnection, or a break with previous roles [5,6].

This individual impact underscores why SUDs represent a global challenge with serious consequences at the individual level. In 2025, there were 51,255 admissions to treatment for psychoactive substance dependence in Spain, representing an increase of 9.7% over the previous year [7]. Moreover, adherence to rehabilitation programs is often limited, with high dropout rates reported internationally and frequently linked to psychosocial vulnerability and difficulties in establishing therapeutic relationships with health care professionals [3].

Beyond physical and mental health consequences, SUDs also affect identity and social functioning, contributing to stigma, social disconnection, and loss of social roles [3]. In Europe, treatment demand continues to rise, with 51,255 admissions for psychoactive substance dependence recorded in 2025, representing a 9.7% increase compared with the previous year [7,8].

Although SUDs affect people of all genders, treatment-seeking populations in community addiction services are often predominantly male, which may lead to the underrepresentation of women’s experiences in qualitative studies [6-8]. This is of relevance given the established role of gender in shaping pathways into substance use, the associated stigma, exposure to violence or trauma, caregiving responsibilities, and help-seeking behaviors. These factors might shape how nursing care is perceived and what forms of support are most beneficial. Therefore, incorporating a gender perspective might be essential when interpreting patients’ accounts and designing nursing interventions in community addiction treatment centers [9].

### Prior Work

Evidence-based rehabilitation programs have been shown to reduce the use of illegal opioids and improve patients’ physical and mental well-being, as well as their quality of life [8]. These programs adopt a holistic approach, with a multidisciplinary team comprising nurses, psychologists, physicians, social workers, and occupational therapists. Furthermore, the centers where these programs are implemented administer substitution treatment and provide social support and nursing care [8,10]. The ideal outcome of substance use rehabilitation is a state of balance and control, where the person feels empowered to manage their life without relying on substances that alter their mental state [7,8]. This process involves a reconstruction of the individual’s identity through a series of behavioral modifications, facilitated by health care professionals [7,9,10]. However, it is estimated that up to 40% of patients withdraw from these programs. In this context, the support and participation of a multidisciplinary team, including nurses, is an empowering element in addressing this condition [11].

The concept of care is understood as a natural phenomenon. The patient’s world, vulnerability, health, and suffering form the core of this concept [12]. The science of care not only facilitates the treatment of disease but also addresses patients’ emotional and social needs, thereby promoting their overall well-being. The quality of care is known to improve when health care professionals make a conscious effort to understand the world from the patient’s perspective. This, in turn, strengthens the therapeutic relationship and promotes better care outcomes. A profound understanding of the experiences and perspectives of patients with SUDs is imperative for the development of patient-centered care. This knowledge facilitates the design of personalized interventions, tailored to the specific needs of this vulnerable population [13,14]. By exploring the subjective experiences of these patients in relation to their care, areas for improvement in health services can be identified, more empathetic and culturally sensitive practices can be promoted, and barriers to access and adherence to treatment can be addressed. This comprehensive strategy for addressing SUDs has the capacity to markedly enhance patients’ quality of life, diminish the social disapproval associated with substance use, and ensure optimal long-term therapeutic outcomes [15].

Despite the presence of nursing in SUD rehabilitation programs, the literature indicates that its role continues to be invisible and poorly understood by both patients and society. In community and outpatient settings, nursing work tends to be diluted within the multidisciplinary team, making it difficult to identify its specific functions [13,14]. However, in addiction treatment centers (CATCs), nurses carry out key interventions that include comprehensive patient assessment, clinical follow-up, health education, administration and supervision of substitution treatments, early detection of relapses, crisis care, and, especially, the establishment of a therapeutic relationship based on trust and continuous support during the rehabilitation process [16]. This apparent contradiction between the relevance of care and the low social recognition of the nursing role justifies the need to explore how users themselves perceive and understand this care [13,17].

### Study Objectives

However, there is a significant gap in the literature on the perception of patients with SUDs regarding the role of nurses in rehabilitation programs [16,17].

The primary aim of this study is to explore the experiences and perceptions of patients with SUDs regarding the nursing care they receive in outpatient and community settings in Spain. Specifically, the study seeks to analyze patients’ perception of the role of nursing staff, considering both the technical and relational dimensions of nursing care as experienced by users and to describe the organizational and care role that users attribute to Red Cross CATCs in Madrid (Spain), including resources, routines, and processes involved.

## Methods

The question of how the role of nursing is perceived in the context of patients with SUDs undergoing rehabilitation programs lends itself to a qualitative approach, specifically a phenomenological study. Qualitative research focuses on understanding the world of the participants [18].

### Study Design

The researchers were interested in how patients recover from SUDs and how they perceive the role of nurses in this process. It was therefore determined that qualitative research would best meet the objective. To gain a deeper understanding of this experience, a phenomenological study was conducted, as in-depth interviews allow the emotional, social, and personal nuances of the rehabilitation process to be captured, as well as the importance of support from nurses [19-21]. This research was conducted based on the recommendations of the COREQ guide [22].

### Participants

Patients diagnosed with SUDs according to the *DSM-5* [1] were selected.

#### Eligibility Criteria

The inclusion criteria established at the beginning of the study were as follows: participants had to be enrolled in a rehabilitation program, be over 18 years of age, and be able to speak Spanish. Patients who did not wish to participate in the study or who were physically and/or mentally unable to complete the interview properly were excluded from the study.

#### Study Sample

A sample of participants was intentionally selected to represent a range of age groups, socioeconomic backgrounds, recovery times, genders, and experiences regarding substance use and the impact of nurses during the rehabilitation process. Furthermore, the data were collated pertaining to the participants’ date of birth, gender, marital status, substance of abuse, alcohol consumption, and number of prior admissions to rehabilitation programs (Multimedia Appendix 1).

Of the 15 participants, 14 were male, and 1 was female. The ages of the participants ranged from 32 to 78 years (Table S1 in Multimedia Appendix 1). The participants’ demographic profile was as follows: 8 were unmarried, 4 were divorced, 2 were married, and none were widowed. The participants consumed a variety of substances, including alcohol, cannabis, cocaine, heroin, opioids, methamphetamines, ecstasy, ketamine, hallucinogens and magic mushrooms, spice, mephedrone, salvia, gamma hydroxybutyrate, and lysergic acid diethylamide. The substances most frequently used were cannabis, heroin, cocaine, and alcohol. The number of previous admissions to rehabilitation programs ranged from 1 to 10. All participants were enrolled in the Red Cross rehabilitation program in Madrid, Spain.

### Access to the Population

Initially, the researchers identified the various support structures for treatment and posttreatment within the Red Cross organization. In-depth interviews were conducted at 2 Red Cross CATCs in Madrid (Spain).

The management team and the multidisciplinary team at the centers were contacted, and meetings were organized to discuss the research objective and obtain the help of the various professionals in identifying participants. Participants were recruited through purposive sampling, following a preliminary presentation of the study to patients at the Red Cross centers together with the center’s management team. Factors that would facilitate a more nuanced categorization of the work were considered, particularly regarding the perception of the nursing role in individual interviews.

### Data Collection

In-depth, individual, semistructured interviews were conducted in person with 15 participants. One participant decided to leave the interview after 14 minutes of recording, so a total of 14 patients completed the interviews. The average interview duration was 45 (SD 15.3) minutes, and the interviews were conducted in convenient settings, such as individual rooms at the CATCs, to ensure privacy. The interviews were conducted between January and April 2023. All interviews commenced with the following opening statement: “Please provide a brief overview of your professional background and the circumstances that led to your involvement in this field.” The flexibility of this qualitative interview methodology enabled participants to share their experiences from their own perspective. Open-ended questions and prompts were strategically used to explore turning points, support, situations, and coping strategies when participants were reluctant to share them. The following questions and prompts were included: “Please find below a list of the key responsibilities that nurses in this institution typically have” (Multimedia Appendix 1). With the prior consent of the participants, the interviews were audio recorded. The data collection protocol for the interviews was consistent for all patients. Interviews were transcribed immediately after completion. Data collection ceased once sample saturation was reached [23].

### Trustworthiness

The criteria used to ensure reliability, in accordance with Guba’s contributions to naturalistic research, encompass credibility, transferability, dependability, and confirmability [24].

#### Credibility

All interviews were recorded, and the exact duration of each session was documented, with additional field notes providing further elaboration and complement.

#### Transference

The processes followed, both for data collection and subsequent analysis, have been described in detail.

#### Dependence

The data collection process and the instrument used for conducting the interview have been delineated and included (Multimedia Appendix 1). Furthermore, members have been added to the working team at different stages to carry out triangulation processes in the negotiation of meanings.

#### Neutrality

The saturation of messages and the inclusion of direct quotes in the final report ensure neutrality in the analysis of the data.

### Analysis

The transcriptions of these recordings were then produced. Subsequently, a comparison was made between the recordings and transcripts to ensure accuracy. Thereafter, a systematic condensation of the text was carried out in the analytical process, with the following 4 steps [25]: first, the transcripts were read to obtain an overview and identify preliminary themes, with annotations made in the text. Second, a series of codes and code groups were developed. In the event of the emergence of divergent codes, the most appropriate ones were agreed upon to develop a shared understanding. Third, each code group was identified and condensed into categories, which were incorporated into the report, with relevant quotes selected for inclusion. Finally, the condensed subgroups were synthesized to generalize descriptions and concepts of the patients’ experiences [26,27]. The data analysis was iterative and began concurrently with data collection, using the constant comparison method as described by Strauss and Corbin [23] to identify commonalities and variations across cases progressively incorporated.

The ATLAS.ti program (version 25.0.1) was used for the analysis, as were the Office suite and Microsoft Excel for descriptive data analysis, and Microsoft Teams for facilitating digital collaboration during coding.

### Ethical Considerations

This study has been approved by the Ethics Committee of the Complutense University of Madrid (Reference: CE_20221215‐06_SAL). Furthermore, the study was conducted in accordance with the principles set out in the Declaration of Helsinki. In addition, to comply with the code of ethics for good practice in the collection of information for this research project, the resources provided by the Complutense University of Madrid have been accessed. The data have been anonymized, with all names appearing in the text being fictitious (Table S1 in Multimedia Appendix 1). Reasonable and appropriate physical, administrative, and technical measures have been taken to protect personal data from loss, misuse, unauthorized access, disclosure, alteration, or destruction. Participants were at liberty to accept or decline participation, and those who agreed to participate signed consent forms. It is important to note that no form of compensation was provided or offered to any of the participants.

## Results

### Participant Characteristics

All participants had received a confirmed diagnosis of SUDs prior to the interview. Of the 15 participants, 14 were male, 8 (54%) were single, 5 (33%) were divorced, and 2 (13%) were married. The mean age of the subjects was 48.2 years (SD 9.28). The substances most frequently consumed were cocaine—used by 13 (86%) of the participants—cannabis and alcohol (Multimedia Appendix 2). The mean number of previous admissions to rehabilitation centers within the sample was 2.5 (SD 2.4).

**Figure 1.**
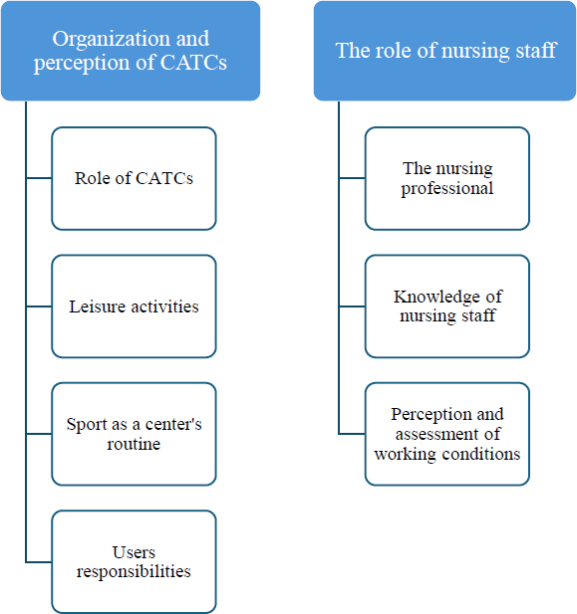
Emerging categories from patient narratives: organization of community addiction treatment centers (CATCs) and the nursing role.

In the following section, the results of the empirical investigation will be presented. The data obtained from the interviews have been organized into 2 overarching categories, which are related to the research objectives and questions (Figure 1). All these categories have been framed from the perspective of the users.

### The Role of CATCs

#### Key Functions of CATCs

The role of Red Cross CATCs is pivotal in helping participants to stop using substances. The interviews revealed several issues. First, these centers play a significant role in helping individuals stop using substances, particularly through measures such as prescribing methadone as a substitute for heroin: “This is how I became acquainted with the CATCs and the process of heroin substitution” (Óscar). It was also noted that the center offers users the possibility to have meals on-site and to receive support with various arrangements, including transport and mobility-related matters. “Additionally, my transport card is being loaded, which is a modest amount of four euros and a few cents” (Óscar).

The same user commented on the existence of these rehabilitation centers. He said that he had gone “to a hospital because I didn’t know that the CATCs existed” (Óscar).

#### Leisure Activities

The importance of leisure activities in the daily lives of participants emerged as a recurring theme in the interviews, with all interviewees highlighting it as a central focus. Javier succinctly summarizes the routine of a typical day at the center, characterizing it as “attending therapy sessions.” This assertion is corroborated by the observations of other participants, such as Oscar, who states: “At the Red Cross therapy centre (...) it helps me a lot because I was busy all day and I didn’t have any nonsense going through my head.” These centers, as a result, organize a variety of activities and “workshops on drugs, gender, reading ...” (Chema), which are emphasized in the interviews. Daniel elucidates the manner these centers provide support:

It’s like, like, like a house where you can’t just sleep. They also make us feel like this is our home. The Red Cross treats us very well and they really love us; they truly love us. I mean, it’s not just a pat on the back and that’s it. They are ... it’s a complete embrace, from heart to heart.

As well as the points raised by Chema, there have been repeated mentions of board games, music, and dance workshops. The latter has been of particular interest, given the work carried out by the volunteer running them:

She herself, who is not like all the other workshops that are all about that, about drugs, about you, about these, about others ... No, she plays music and says, “Now you do whatever you want“ and ”Now you dance, and now you ... and now everyone together here and now everyone there ... now everyone ...” I mean ... she makes you forget everything at that moment. No, you don’t think about your problems, you don’t have time to think about anything bad, or anything that could hurt you, that could ... She doesn’t give you time, she doesn’t let you. She’s full of life.

[María]

#### Sport in the Center’s Routines

Sport has also been mentioned in numerous interviews, often referred to as “sports activities in the morning” (Paco). To that end, the following activities have been outlined:

- “Playing table tennis or cycling or exercising.” (Rodolfo)
- “Some days it’s Tai Chi, other days they go walking.” (Maria)‬‬‬‬
- “I played volleyball, which I hadn’t played in years.” (Roberto)‬‬‬‬‬
- “Now we are cycling.” (Antonio)

In addition, sport has been referenced on a single occasion as a concomitant treatment for other conditions: “I come walking to get some exercise because of my diabetes” (Chema).

#### Users’ Responsibilities in the Centers

Leisure time is also spent carrying out different responsibilities assigned by the center:

Oh, and if it’s your turn to do the office work, you have to set the table. If it’s your turn to wash up, you have to wash up too, and so on.

[Fulgencio]

“As for routines ... we have a plan too ... there’s a ... a notebook where they write down when we have to prepare meals, wash the dishes ... That’s how we keep busy. .. the manager of the centre writes it down for everyone. And on days when it’s my turn, it’s someone else’s turn ... When it’s my turn, I have to serve the tables, then clear the dishes, take out the rubbish bins ... and so on, routine stuff ... To clean the dishes, then dry them ... That’s how it works, I don’t know, to keep us busier.”

[Óscar]

Then one day it’s your turn to wash the dishes and another week you’re in charge. You’re responsible for distributing the food and so on, but hey, they’re supervising you.

[Rodolfo]

### Functions of the Nursing Professional

#### Role of Nurses in CATCs

A variety of professionals are employed in these centers, including nurses, doctors, psychiatrists, psychologists, and occupational therapists. Nurses have been the central focus of the work, and they have taken up a large part of the interview time. The functions of nursing staff are those that have been most frequently mentioned in the coding process, in relation to the perception of this group. It is generally understood that the profession is held in high regard by its users. There are some very compelling testimonials in this regard:

At the Jiménez-Díaz Foundation, nurses treat you excellently. I can’t say anything bad about healthcare here in Spain or about the nurses, they are all lovely and very professional.

[Roberto]

These comments are not isolated, as they are reinforced by other comments that echo the same sentiment.

For example, Roberto stated:

The nurse who works with the doctor [at the CATC] (...) is a lovely girl, she is super friendly, she is a sweetheart. Professionally, she is excellent, as they say. And also, she was very concerned and asked me: are you okay?

Daniel also commented that “the treatment by the nursing staff [at the CATC] was very good, very good, very good.” He particularly highlighted the treatment by the nurse above all others. When asked about the care that had meant the most to him, he pointed to the emotional support provided by a nurse:

I’m very clear about this. It’s (...) a nurse I had at the shelter [at the CATC] (...), accompanying me to an ambulance because I have tinnitus. Then they were saying bad things to me, and she was the first person I talked about it. And that, and how she accompanied me and went down the stairs with me ... I was shaking because I didn’t want to go to the ambulance, I didn’t want to go there. I was terrified of being alone for even a second with the voice. And that’s the most the nurses have done for me.

[Daniel]

On occasion, this positive perception was intertwined with the work of the entire team, as Antonio noted:

Here [at the CATC] we have a psychologist, a social worker, a nurse, the manager, very good people, the workers too. They get angry, which is normal sometimes, but they are people.

The testimonies are intricately interwoven throughout the interview, thereby underscoring the human dimensions of the nursing professional. Various statements highlight the staff’s attentiveness and their provision of empathetic listening and support, in response to the most useful care they have received:

Therapies where you can talk, like two people. You know what I mean? Being able to sit down, two people. You, with a therapist, being able to sit down and have .. him being able to tell you about his problems and you being able to tell him your problems, having someone there to talk to unburden yourself. Telling him, “Look, I got out of bed and cried.” I cried because of this, because of this and because of this, and being able to tell you ... your things and him being able to tell you his things.

[Jorge]

This girl … the girl on the other side, at the Red Cross [at the CATC]. She ... I saw that she was concerned about my situation the first day I went there. I ... I thought she was a psychologist rather than a nurse, from the time I spent talking to her and expressing my feelings, my experiences, where I came from, where I was going, and why I found myself where I was. I was there for, I don’t know, two hours, three hours, talking to her, and she was a nurse, not a psychologist. And she was concerned about ... about how I was doing.

[Jero]

Even when pointing out other functions, Paco considered that

the way the staff [at the CATC] treat you is what matters most ... because that’s what affects you the most. But then, of course, each person ... the pills and so on ... also have an influence. But ... the way they treat you is what attracts you the most.

Rehabilitation programs were also identified as particularly valuable forms of care, with participants citing the significance of being listened to in difficult situations:

My nurse [at the primary care centre], who has helped me to trust her a little, weigh myself, tell her when I have used drugs and when I haven’t, give thanks, I am grateful to the people who have been helping me since 2015. And even though I messed up and stuff, they did everything they could to help me (...) see what we needed.

[Óscar]

In other words, the same patient insisted:

She was teaching nursing classes here, for everyone. That was very positive because there were classes on all kinds of illnesses, how to use pills ... There were also classes on quitting smoking, how to quit smoking ... There are workshops for people who want to quit smoking too. These workshops are run by nurses. That’s all I can say for now because I can’t remember anything else.

[Óscar]

María was very clear on this point: “They [at the primary care center] ... do a bit of psychology and nursing, kind of combining the two.” Another user, Jero, who had already pointed out the importance of nursing staff’s ability to listen, emphasized the importance this can have in other therapeutic aspects, such as methadone use:

Well, if this is the first day—I said—“it shouldn’t take too long.” He [at the CATC] offered me something ..., which was methadone, and I said, “Look, I don’t want that, I’ve never taken that in my life, but I’m afraid, because I’ve heard that there are people who spend their whole lives taking that crap.” I said, “Well, let’s start with a very, very small amount. Let’s see if that’s enough for you and then we’ll reduce it.” “Okay, I’ll accept it, because of the way you’ve treated me, because I can see that you’re someone who cares about the situation I’m in without knowing me at all, and because I’ve ... I’ve opened up to you and told you about ... my plans.” It was really good, to be honest, that’s the part I’m most grateful for.

[Jero]

#### Knowledge of Nursing Staff

During the interviews, references to the work of nurses were particularly prevalent, especially in discussions of more general topics and when the work functions of the entire team were intertwined. There are numerous examples of this phenomenon, particularly in relation to the role of the doctor. For instance, when asked about the duties of a nurse, Paco replied:

Well, we ... I, for example, go up to cognitive workshop, which was today ... to the girl in cognitive workshop [at the CATC]. And then, in case you have ... there’s the doctor, the doctor in case something happens to you. I have a toothache and I ask him for ... a pill for toothache because it hurts and I still haven’t ... I go to the medical centre by bus and I always forget to stop at medicine.

Another patient intermingled the roles of different professionals in his reflections:

I haven’t had much … much … much experience with nurses, because as I’m new, I haven’t had much time. What I do have is a good day centre, which I’ve had for two days now, OK? And I only have experience with Susana, who is my psychologist, and with Dr. Felipe, I don’t know if you know him. They are the only ones I have experience with.

[Roberto]

Comparisons were even drawn between nurses and doctors:

You know more. You are not doctors, but we can trust you more than doctors. I am quite bold, having been a salesperson all my life, so I don’t mind, but ... I think a doctor can be more reserved than a nurse.

[Eduardo]

These relationships and role confusions are not exclusive to CATCs, as they also mentioned past experiences in which they highlighted the importance of nurses in primary care centers:

At the mutual insurance company, for example, this leg that I told you I hurt while cleaning ambulances, they treated me like a queen. Both in physiotherapy and in ... when it came to medication, creams, everything, and all for free.

[María]

Not everyone knows about nursing. There are things that if you have, if you have a wound here, you put Betadine on it or whatever and that’s it. But there are things that they have to give you. They have to tell you, “Look, the stretch consists of squats, this, that and the other, OK?” (...) Well then, I think that time should also be used to ... I see that they devote a lot of time to older people [at the primary care centre].

[Maria]

#### Perception and Assessment of Working Conditions

Another noteworthy aspect that emerged from the interviews was related to working conditions, including issues of overburden:

Well, I can see that he’s a bit overworked, but even so, he’s always available [at the CATC]. Yes, if I could add something ... a little help, give him some assistance. Because he does tests, dispenses medication, sees patients ... You don’t have to make an appointment with him. Because you talk to him beforehand and Julián is immediately available.

[Chema]

Appointments take a long time. For example, those at the public centre and at the outpatient clinics too. Sometimes they can’t see you, even if it’s an emergency, because they have someone else who maybe ... well, you say it yourself ... I mean, I don’t pay for my medication, do I? Because I earn very little. So there are times when I’ve had to buy the medication because I needed it at that moment because they don’t have enough nurses [referring to nurses in outpatient/primary care consultations]. They keep cutting back instead of ... providing more for the population.

[María]

This same patient, María, concluded very clearly: “I think people should be given more permanent jobs.”

## Discussion

### Principal Results

Patients with SUDs perceive nursing care positively, although they predominantly highlight relational aspects, such as accompaniment, active listening, and trust. In some cases, there appears to be a lack of awareness regarding nursing functions, particularly those within the scientific and technical domains. The results of this study provide valuable insight into how patients with SUDs value and interpret the nursing care they receive in outpatient and community-based rehabilitation programs in Spain.

From a phenomenological perspective, understanding patients’ perceptions of the role of nursing requires first delving into their personal history of substance use. This is not merely a chronological account of events; it is a complex network of lived experiences, emotions, social relationships, and meanings attributed to the substance and its effects [28]. The mean number of previous admissions to rehabilitation centers in the sample was 2.5 (SD 2.4). This aspect also frequently features in patients’ accounts, highlighting the chronic nature of SUDs and the difficulties in achieving sustained rehabilitation over time [4,7,28].

Participants offered a largely favorable evaluation of the center, regarding it as a vital asset in their rehabilitation process. This positive perception appears to be associated with the multidisciplinary team, comprising doctors, psychologists, social workers, occupational therapists, and nurses. The team’s role is to address both the physical and psychological aspects of treatment and provide comprehensive support that extends beyond a purely clinical approach [12,13,15]. In their testimonies, participants highlight the professional and emotional support they received, as well as the feeling of belonging to a safe and structured environment that not only distances them from problematic consumption environments but also provides for their basic needs and offers opportunities for improvement in various areas.

On the other hand, participating in a variety of group activities, such as physical exercise, healthy leisure initiatives, and workshops focusing on integrating patients into the workplace and fostering personal relationships, was considered vital for sustaining abstinence. Physical exercise is widely used as a complementary therapy in SUD treatment. It is considered a healthy practice, as well as a meaningful and socially integrative activity [29,30]. Similarly, the other leisure workshops offered at the center are seen as potentially transformative by providing entertainment in environments unrelated to substance use and fostering a sense of belonging to a social group [28,29]. One possible explanation for these findings is that the center is perceived not only as a protective factor against relapses, but also as a safe resource for well-being and ongoing support. This finding is consistent with other studies addressing this issue but contrasts with previous studies documenting experiences of stigmatization and discriminatory treatment in hospital settings. The role of the multidisciplinary team could be pivotal in generating a positive overall experience that encourages adherence to the rehabilitation program and social integration [30,31]. This approach is consistent with the Schlossberg transition process model, in which the authors recommend that, to effectively cope with a transition—namely, rehabilitation from SUDs—people need access to diverse types of support [32]. These support categories can range from interpersonal relationships (such as sponsors and spouses) to more distant support systems (such as institutions and communities), including rehabilitation centers [33]. Consequently, rehabilitation programs that adopt a holistic perspective—combining respect for patient autonomy with a flexible and adaptable support structure—align not only with international standards but also with a patient-centered approach [7,10].

Regarding nursing care, the participants in our study recognize nurses as key figures in the rehabilitation programs of the Red Cross CATCs. Most of the nursing practices identified as beneficial by the participants in this study pertain to relational care interventions. They emphasize their close working relationships, active listening skills, and emotional support as key differentiating factors compared to other health care professionals in the multidisciplinary team. Furthermore, they place a high value on the involvement of nursing staff, particularly in health education, support in times of crisis, and management of withdrawal symptoms. This finding aligns with the results of other studies, which indicate that patients with SUDs perceive increased support and reduced judgment from nursing professionals [34,35]. As demonstrated in previous research, the provision of compassionate care is of paramount importance, even in relation to other populations [36,37].

### Comparison With Prior Work

This evidence indicates that the therapeutic relationship is fundamental for patients, regardless of their age or care setting. Skills, such as listening, communicating, and providing reassurance, are valued and recognized as essential, not as inferior or merely technical skills [38]. In addition, other studies have shown that relational interventions generate a wide variety of positive health outcomes. Research has demonstrated that effective therapeutic relationships can enhance treatment adherence and rehabilitation and reduce perceptions of stigma, as outlined in Parkhideh et al [39]. As demonstrated in the literature and as evidenced by our own findings, there is clear evidence of the importance that patients attach to being treated with respect and as people rather than as problems or diseases [40].

Among the recommendations expressed by users, the one that stands out is the suggestion that health care personnel show greater patience during care. This demand reflects a need for more humane and empathetic treatment and highlights the importance of the therapeutic relationship component. Patience is seen as a sign of understanding, respect, dignity, and a willingness to listen, which are all basic elements in building an effective working relationship. This is particularly relevant in contexts involving vulnerable populations, such as individuals with SUDs. Integrating this perspective into clinical practice has the potential to enhance the quality of care and ensure adherence to treatment [35,38].

However, there is a lack of knowledge regarding the roles performed by each member of the health care team. Although the role of the nurse remains constant within the team, participants confuse their role with those of doctors and social workers during the discussions. This is also evident from the absence of any codes or categories relating to other areas of nursing care. One potential explanation for these findings is that patients may not have had sufficient time to comprehend the nature of nursing work. However, it should be noted that all participants had previously been admitted to at least 1 other rehabilitation program. Therefore, the lack of knowledge about nursing work may not be attributable to a lack of contact with nursing staff, but rather to the invisibility of care. The extant literature supports this assertion. In both this population and the general population, care is often not directly associated with nursing. This may mean that the specific and specialized role of nurses in various contexts is not fully recognized. This lack of knowledge about nursing care reflects an underlying problem, which contributes to its social and professional devaluation [41,42].

The participants’ accounts also allow us to infer a structural reality that affects the professional practice of nursing: the low institutional and social value placed on this group. This situation is evident in working conditions that often fail to reflect the complexity of the care required by patients with SUDs. These conditions include a lack of financial recognition, excessive workloads, a shortage of adequate rest areas for staff, and limited participation by nurses in decision-making spaces. This invisibility stands in stark contrast to the pivotal role that patients themselves ascribe to nurses in their recovery processes, particularly regarding human support, active listening, and the establishment of long-term therapeutic bonds [43]. This structural invisibility might help explain why some participants were uncertain about the specific responsibilities of each professional, occasionally mixing up nursing functions with those of other team members. This confusion may also arise from overlapping interdisciplinary tasks, inconsistent role introductions—particularly at admission or during care-plan changes—and contextual factors in SUD care, such as time pressure, staff turnover, and psychosocial vulnerability. Therefore, strengthening role communication and increasing the visibility of nursing responsibilities might enhance patients’ understanding and recognition of nursing care within community addiction services [41]. Role ambiguity may also limit patients’ ability to actively seek nursing support. Brief standardized introductions at admission, visible role identification, and, when feasible, assigning a reference nurse may improve clarity. Future research should further explore patients’ perspectives on strategies to enhance understanding of nursing roles in care [41,44].

### Limitations

This study is one of a limited number of research projects conducted in Spain that address the perceptions of a particularly challenging-to-reach demographic, such as patients with SUDs. However, it is vital to understand the experiences of individuals with SUDs and their interactions with nursing care in order to improve and personalize interventions and establish effective improvement strategies within this particularly vulnerable population. Nevertheless, this study presents some limitations. Most of the participants in the sample are male, and most of them self-identify as such. This finding is consistent with the available evidence, which points to a higher prevalence of substance use among men [2]. From a gender perspective, this pattern may be influenced by social constructs associated with masculinity. These constructs include the normalization of risk, pressure to demonstrate strength or autonomy, and perceived lower access to emotional support networks [4]. These factors may facilitate the initiation and maintenance of use and therefore bias the findings. Conversely, the participants were members of merely two centers belonging to the same rehabilitation Red Cross program, which may have exerted an influence on the results due to the absence of variety in the care received. Furthermore, no distinction was made between different types of nursing care (specialized, hospital-based, or based on the years of experience of the professionals). To expand the knowledge base regarding this population’s perceptions of the nursing role, future studies involving samples of patients from diverse rehabilitation programs and incorporating a gender perspective are essential. Women’s perspectives may be insufficiently captured in this study, and future research should purposively recruit more women (and gender-diverse participants when possible) to better inform gender-responsive nursing care in community addiction services.

### Conclusions

This study demonstrated that SUD is a chronic condition, with multiple admissions to rehabilitation programs. Participants have provided a positive evaluation of the rehabilitation center, emphasizing the comprehensive support provided by the multidisciplinary team and the safe environment it offers, beyond clinical treatment. Group activities and ongoing support are key factors in maintaining abstinence and promoting social integration.

Patients with SUDs place a high value on the role of nursing, particularly relational interventions over technical interventions. However, this assessment contrasts with a lack of knowledge about certain functions of the nursing profession, which limits their ability to recognize and take advantage of their capacity for action in community settings.

These findings emphasize the necessity to enhance care models through a holistic approach, considering patients’ life contexts and recognizing both individual care and structural conditions. Nurses can play an essential role in designing patient-centered interventions to achieve sustainable recovery and a positive therapeutic experience.

## Supplementary material

10.2196/82401 Multimedia Appendix 1 Sociodemographic data, participant information, and interview guide.

10.2196/82401 Multimedia Appendix 2 Most used substances among interviewed participants enrolled in the rehabilitation program.

## Abbreviations

CATC: community addiction treatment center
*DSM-5*: *Diagnostic and Statistical Manual of Mental Disorders, Fifth Edition*
SUD: substance use disorder
