# Supplementary material for: Patients’ Perceptions of the Role of Nursing in Substance Use Disorder Treatment Programs: Qualitative Study
Source: JMIR Nurs. 2026 Mar 31;9:e82401. doi: 10.2196/82401 (PMC13038182; doi:10.2196/82401)
Supplement: Multimedia Appendix 1 [file nursing-v9-e82401-s001.docx]

## Multimedia Appendix 1

## Additional file 1

**Fecha de nacimiento**:

**Sexo**: Hombre ❑ Mujer ❑ Prefiere no contestar ❑

**Estado civil:** Soltero ❑ Casado ❑ Divorciado ❑ Viudo ❑

**Tipo de sustancia(s) consumida (s):**

Cocaína ❑ Heroína ❑ Éxtasis ❑

Analgésicos opioides ❑ Cannabis ❑ Anfetaminas ❑

Alucinógenos y setas mágicas ❑ Metaanfetaminas ❑ Esteroides anabolizantes ❑

Ketamina ❑ Spice ❑ Mefedrona ❑ Salvia ❑ Ayahuasca ❑

Otros: _________________________________________________

**Consumo de alcohol:** ❑ No ❑Sí Cantidad a la semana: ______________

**Número de ingresos previos en centros de deshabituación:** ______________

1) **Bloque temático 1**: historia de consumo: “*Háblame de ti” “¿Cómo empezaste con esto?” “¿Por qué consumes?” “¿Qué te hace?”, “¿Qué te provoca?”*

2) **Bloque temático 2**: historia del proceso de deshabituación: *“¿Qué es lo que haces en un día normal aquí?”*

3) **Bloque temático 3**: percepción del trabajo de enfermería: *“¿Qué tipo de cosas realizan aquí las enfermeras?”, “Si pudieras dar un consejo a las enfermeras para que ayudaran a un compañero o a ti, ¿Cuál sería?” “De todos los cuidados que has recibido ¿Cuál ha sido el más útil? ¿Qué ha significado para ti?”*

## Additional file 2

**Table S1. Participant Information (Pseudonyms and Age).**

|  | **Nombre** | **Age** |
| --- | --- | --- |
| **1** | Óscar | 47 |
| **2** | Chema | 49 |
| **3** | Francisco | 38 |
| **4** | Javier | 52 |
| **5** | Fulgencio | 46 |
| **6** | Antonio | 51 |
| **7** | Marcos | 42 |
| **8** | Eduard | 59 |
| **9** | Roberto | 61 |
| **10** | Daniel | 28 |
| **11** | Jero | 29 |
| **12** | Jorge | 32 |
| **13** | María | 53 |
| **14** | Rodolfo | 29 |
| **15** | Paco | 34 |
