# Supplementary figures and images for: Patients’ Perceptions of the Role of Nursing in Substance Use Disorder Treatment Programs: Qualitative Study
Source: JMIR Nurs. 2026 Mar 31;9:e82401. doi: 10.2196/82401 (PMC13038182; doi:10.2196/82401)

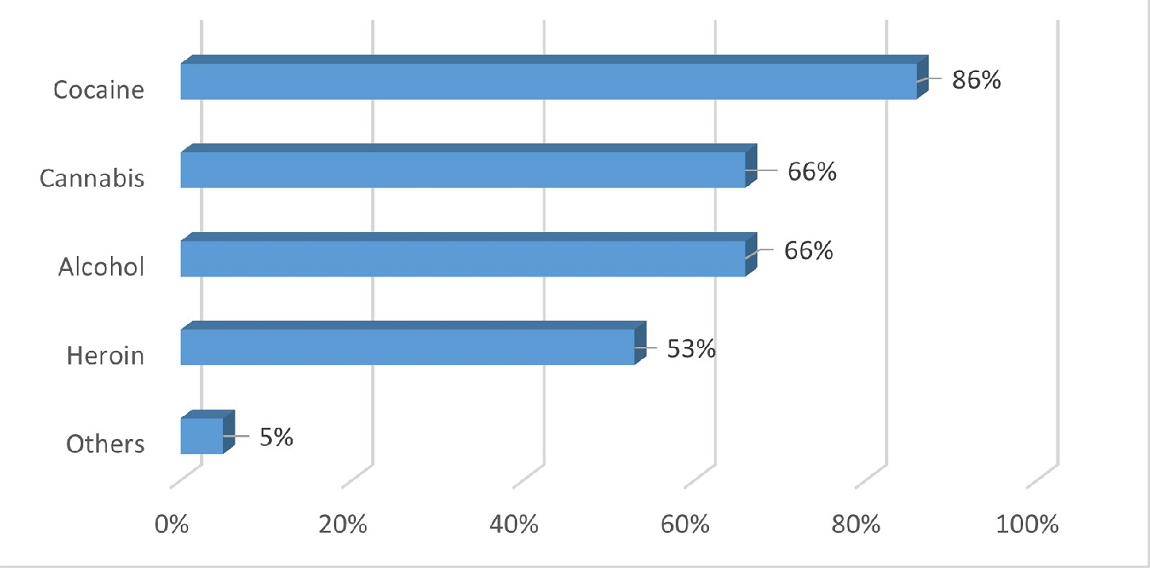

Supplement: Multimedia Appendix 2 [file nursing-v9-e82401-s002.png]
